# The Association between Autism Spectrum Disorder and Precocious Puberty: Considering Effect Modification by Sex and Neuropsychiatric Comorbidities

**DOI:** 10.3390/jpm14060632

**Published:** 2024-06-13

**Authors:** Yi-Chun Liu, Yin-To Liao, Mei-Hong Wen, Vincent Chin-Hung Chen, Yi-Lung Chen

**Affiliations:** 1 Department of Psychiatry, Changhua Christian Children’s Hospital, Changhua 500, Taiwan; 183711@cch.org.tw; 2 Department of Psychiatry, Changhua Christian Hospital, Changhua 500, Taiwan; 3 Department of Healthcare Administration, Asia University, Taichung 413, Taiwan; 4 Department of Psychiatry, China Medical University, Taichung 404, Taiwan; je2tezy@yahoo.com.tw; 5 Department of Psychiatry, China Medical University Hospital, Taichung 404, Taiwan; 6 Department of Pediatric Endocrinology, Sing Wish Hospital, Kaohsiung 813, Taiwan; m75031@gmail.com; 7 School of Medicine, Chang Gung University, Taoyuan 333, Taiwan; cch1966@gmail.com; 8 Department of Psychiatry, Chiayi Chang Gung Memorial Hospital, Chiayi 613, Taiwan; 9 Department of Psychology, Asia University, Taichung 413, Taiwan

**Keywords:** autistic spectrum disorder (ASD), precocious puberty, effect modification, relative excess risk due to interaction (RERI)

## Abstract

Limited knowledge is available about the association between autistic spectrum disorder (ASD) and precocious puberty. Our study examined the association between the two medical conditions and effect modification by sex and neuropsychiatric comorbidities in a nationwide population. To compare the risk of precocious puberty between ASD and non-ASD cases, we conducted a Cox regression analysis using ASD as the exposure and time to precocious puberty as the outcome. We adjusted for sex, attention-deficit/hyperactivity disorder (ADHD), tic disorder, obsessive–compulsive disorder (OCD), anxiety disorder, intellectual disability, and epilepsy. We performed a moderation analysis to examine the potential moderating effects of sex and comorbidities. Patients with ASD were prone to have precocious puberty, with an adjusted hazard ratio (aHR) of 1.80 (95% CI: 1.61–2.01). For effect modification, sex, specifically females, moderated the association between ASD and precocious puberty, with a relative excess risk due to interaction (RERI) of 7.35 (95% CI 4.90–9.80). No significant effect modification was found for any of the comorbidities within the scope of additive effect modification. We found that patients with ASD were prone to precocious puberty, regardless of sex or comorbid neuropsychiatric disorders. Girls with ASD are at a particularly higher risk of developing precocious puberty.

## 1. Introduction

Precocious puberty is a female-dominant disease characterized by the premature development of secondary sexual characteristics, which occurs before the age of nine in males and eight in females [1]. The incidence of precocious puberty, especially central-type precocious puberty, is on the rise [2,3], and the reasons remain unknown. The prevalence of precocious puberty varies according to the region and sex. In Denmark, the prevalence is less than 0.05% in boys and 0.2% in girls [4]. Its prevalence is approximately 0.11% in boys and 4.11% in girls in Korea [5]. The female-to-male ratio is between 4 and 38. According to its etiology, precocious puberty can be divided into two categories, central and peripheral, with the central type being more common [6]. However, the etiology of most central-type cases is unknown [7]. Only very few cases can be traced back to associations with rare genetic mutations or congenital brain abnormalities [7]. Precocious puberty has a significant impact on children’s mental health [6] and may seriously affect their height in adulthood [8]. The early identification of vulnerable groups at risk of precocious puberty can facilitate early intervention for these children, leading to improvements in their quality of life.

Autism spectrum disorder (ASD) is a male-predominant neurodevelopmental disorder characterized by rigidity of behavior and poor social cognitive function [9]. The global prevalence rate is 1% and continues to rise [10]. The male-to-female ratio of this disorder is 4–5 [10]. It has been recognized that ASD is a heterogeneous disorder [11]. More than 70% of ASD cases are comorbid with other neuropsychiatric disorders, such as intellectual disability, epilepsy, attention-deficit/hyperactivity disorder (ADHD), tics, obsessive–compulsive disorder (OCD), and anxiety disorder [12,13,14,15]. ASD that is comorbid with specific neuropsychiatric disorders may suggest a unique pathophysiology that differs from ASD alone [16,17]. In our review, sporadic case reports documented precocious puberty in patients with ASD [18,19]. However, large-scale studies on the association between these two medical conditions remain scarce. Only one cohort study by Geier et al., who utilized the electronic database from the Florida Medicaid system, reported a significantly increased incidence of precocious puberty in children with ASD, with an adjusted hazard ratio (aHR) of 4.64 [20].

Based on the rare studies that have examined the association between ASD and precocious puberty, several issues need to be addressed in detail. First, Geier et al. did not address the influence of neuropsychiatric comorbidities. Some comorbidities, such as ADHD and epilepsy, have been reported to be associated with the risk of precocious puberty [21,22,23]. These comorbidities are often comorbid with ASD, with a complex etiology [16,17]. Therefore, the possible confounding and moderating effects of neuropsychiatric comorbidities on this association should be further investigated. On the other hand, the female-to-male ratio is significantly different between ASD and precocious puberty. Thus, the influence of sex on the association is a topic of interest but cannot be extrapolated from their results. Finally, the source population adopted by Geier et al. was the Medicaid system in the United States. This insurance system covers specific populations, a substantial proportion of whom are immigrants [24]. Children from immigrant families have been reported to be at a higher risk of ASD [25] and precocious puberty [26]. The results from this specific population may cause selection bias, thus limiting their generalizability.

To fill these research gaps, we examined the association between ASD and precocious puberty in a representative nationwide population. More importantly, we investigated the moderating effects of different neuropsychiatric comorbidities and sex on the association between ASD and precocious puberty. We conducted a retrospective cohort study to examine the association and effect modification.

## 2. Materials and Methods

### 2.1. Data Source and Study Design

**Figure 1:**
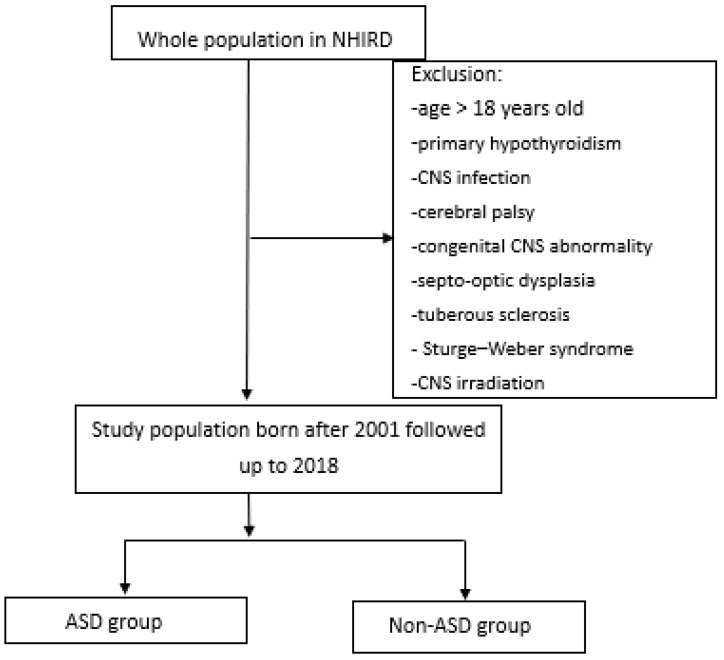
The flowchart of the study. ASD, autistic spectrum disorders; CNS, central nervous system.

Since 1995, Taiwan has had a single-payer National Health Insurance (NHI) program. According to an official statement in December 2021, the program currently covers 99.9% of the whole population in Taiwan [27]. The use and analysis of the NHIRD do not require informed consent because the dataset was de-identified and delinked, making it impossible to trace back to individual participants. Our study data were obtained from de-identified enrollment data from the National Health Insurance Research Database (NHIRD) [27]. These data included hospitalizations, outpatient clinic visits, emergency room visits, drug prescriptions, visit times, and diagnosis codes [28]. We designed a retrospective cohort study that utilized the entire population data between 1 January 1997 and 31 December 2013. The flowchart of our study design is presented in Figure 1.

### 2.2. Study Population

The inclusion criterion was individuals born between 1 January 1997 and 31 December 2010. The study follow-up period was from the date of birth until the index date of precocious puberty, death, or 31 December 2018, whichever occurred first.

The exclusion criteria for the study were other organic factors that may contribute to precocious puberty based on previous studies [21,29], including the following: primary hypothyroidism, central nervous system infection, congenital abnormalities of the central nervous system, septo-optic dysplasia, tuberous sclerosis, Sturge–Weber syndrome, and ever radiation to the central nervous system. Individuals with these diseases were excluded. The above diagnoses were coded based on the International Classification of Diseases, Ninth Revision, Clinical Modification (ICD-9-CM) and ICD-10 (Supplementary Table S1).

### 2.3. Exposure Variables and Study Outcome

We identified ASD cases if they had at least one medical visit with an ICD9-CM of 299 or an ICD-10 of F84 during the study period. The earliest date of a diagnosis code for ASD was designated as the index date of ASD. For the sensitivity analyses, we used a more stringent set of diagnosis codes to define ASD, such as 299.0 for ICD9-CM and F84.0 and F84.5 for ICD-10.

The outcome was defined as a diagnosis of precocious puberty with an ICD9-CM of 259.1 or an ICD-10 of E30.1 on at least one medical visit during the study period. Similarly, the earliest date on which a diagnostic code for precocious puberty was recorded was designated as the index date of precocious puberty.

### 2.4. Effect Modifiers

We listed sex and several neuropsychiatric comorbidities as potential effect modifiers. These comorbidities include ADHD, tics, OCD, anxiety disorder, intellectual disability, and epilepsy. We defined a person as having one specific comorbidity if that comorbidity diagnosis had been documented on at least one medical visit during the study period. The comorbidities and their diagnostic codes were as follows: ADHD (ICD9-CM 314 and ICD-10 F90), tics (ICD9-CM 307.2 and ICD-10 F95), OCD (ICD9-CM 300.3 and ICD-10 F42), anxiety disorder (ICD9-CM 300.0 and ICD-10 F41.0, F41.1, F41.9), intellectual disability (ICD9-CM 317, 318, 319 and ICD-10 F70, F71, F72, F73, F78, F79), and epilepsy (ICD9-CM 345 and ICD-10 G40, G41).

### 2.5. Covariates

In the main analysis, we evaluated the association between ASD and precocious puberty. Thus, sex, neuropsychiatric comorbidities, and low-income households were set as covariates. The definition of a low-income household was that after the monthly household income was evenly distributed to each family member, the average monthly income per member was less than the monthly minimum living expense of that residence region [30].

### 2.6. Statistical Analysis

We used the Cox proportional hazard model with age as the time scale to analyze the survival data in our study. A preliminary analysis was performed, and the proportional hazards assumption was tested using ln(₋ln) plots and Schoenfeld’s residual tests. Only the sex variable violated the assumption. Therefore, the sex variable was controlled for through stratification. To assess the association between ASD and precocious puberty, we conducted Cox regression with ASD as the exposure and time to precocious puberty as the outcome. We also adjusted for sex, ADHD, tics, OCD, anxiety disorder, intellectual disability, and epilepsy in the analysis.

We also performed a moderation analysis to examine the potential moderating effects of sex or neuropsychiatric comorbidities on the association between ASD and precocious puberty. We conducted the moderation analyses as suggested by Knol and VanderWeele [31]. Here, we set ASD as the exposure, time to precocious puberty as the outcome, and sex and neuropsychiatric comorbidities as the potential effect modifiers. First, we categorized the study population into four strata based on the exposure (ASD) and each effect modifier (sex or specific neuropsychiatric comorbidities). We then calculated the aHRs with 95% confidence intervals (CIs) for precocious puberty stratified by ASD and each effect modifier. There was only one single reference group. For example, when considering effect modification by sex, we used the male non-ASD subgroup as the reference. When considering effect modification by neuropsychiatric comorbidities, we used the non-comorbidity and non-ASD subgroup as the reference. Second, we calculated aHRs with 95% CIs for precocious puberty within strata defined by each effect modifier. For example, to examine the sex-specific associations of precocious puberty, we categorized the study population into two strata (males and females) and then calculated aHRs with 95% CIs for precocious puberty in each stratum. Finally, we determined the measures of effect modification on both additive and multiplicative scales. The additive scale used in this study is the relative excess risk due to interaction (RERI) [32]. The 95% confidence intervals (CIs) for the RERIs were calculated with reference to the previous literature [33]. A RERI not equal to zero indicates that the association of ASD with precocious puberty varies by strata defined by sex or comorbidities, which has been referred to as effect heterogeneity [34]. The additive effect modification is deemed to be positive if RERI is greater than zero and negative if RERI is less than zero. A positive RERI suggests that the association of ASD with precocious puberty was more pronounced in females or in patients with specific comorbidities. The same rationale can be applied to explain a negative value of RERI. On the other hand, to obtain the multiplicative effect modification, we added cross-product terms between exposure and effect modifiers to the multivariable Cox regression model [35]. The multiplicative effect modification is deemed to be positive if the ratio is greater than one and negative if the ratio is less than one. Positive values on the ratio scale indicate that the combined effect of ASD and the effect modifier (i.e., sex or specific neuropsychiatric comorbidity) outweigh the product of the effect of ASD and the effect modifier. The same rationale can be applied to explain a negative value of the ratio.

## 3. Results

### 3.1. Demographics and Incidence

Initially, 3,387,576 children born between 1997 and 2010 were included in the study. After applying the exclusion criteria, 3,342,077 patients remained in the study. Of these, 29,320 were diagnosed with ASD and 3,312,757 were controls. Table 1 presents the basic demographic data of the study population. In the ASD group, there was a male predominance with a ratio of 5.54. If we applied a stringent definition of ASD, i.e., ICD9-CM 299.0, ICD-10 F84.0, or ICD-10 F84.5, there were still 24,102 people who met the diagnostic criteria for ASD. The prevalence of comorbidities was much higher in the ASD group than in the non-ASD group, especially ADHD diagnoses, where up to 64.12% of individuals with ASD suffered from this comorbidity. The rate of precocious puberty was 1.20% and 0.94% in the ASD and non-ASD groups, respectively.

### 3.2. Incidence of Precocious Puberty in ASD

As shown in Table 2, patients with ASD have a significantly higher risk of precocious puberty than non-ASD patients, with an adjusted hazard ratio (aHR) of 1.80 and a 95% CI of 1.61–2.01. The result held when ASD was more strictly defined, with an aHR of 1.86 and a 95% CI of 1.64–2.09.

### 3.3. Moderation Analysis of Sex between ASD and Precocious Puberty

The results of the moderation analysis are listed in Table 2. In either the male or female cases, individuals with ASD were more likely to experience precocious puberty than those without ASD, with aHRs of 1.69 and 1.86, respectively. There was a positive RERI of 7.35 with a 95%CI of 4.90–9.80 in the moderation analysis, which implies a synergistic effect modification by females on the association between ASD and precocious puberty. However, the multiplicative effect was not significant.

### 3.4. Moderation Analysis of Comorbidities between ASD and Precocious Puberty

The results of the moderation analyses with each of the six comorbidities as an effect modifier are presented in Table 3. We observed that ASD was significantly positively associated with precocious puberty in all comorbidities, with aHRs ranging from 1.57 to 2.81. Although we observed ADHD as an effect modifier on the association between ASD and precocious puberty on the multiplicative scale (aHR 0.67, 95% CI 0.42, 1.05), this moderating effect was not replicated on the additive scale. No significant effect modification was found for any other comorbidity on either the additive or multiplicative scale.

## 4. Discussion

To our knowledge, this is the first study to investigate the association between ASD and precocious puberty in an Asian population and also the first to examine the effect moderation by sex and neuropsychiatric disorders on this association. We found that patients with ASD were prone to precocious puberty in life, regardless of sex or comorbid neuropsychiatric disorders. Additionally, we found that only sex modified the association between ASD and precocious puberty from the perspective of the additive scale, but not neuropsychiatric comorbidities. In other words, female patients with ASD are at a particularly higher risk for precocious puberty. These findings alert clinicians to pay more attention to the timing of pubertal development in patients with ASD, especially girls with ASD.

Our results are consistent with those of previous studies [18,19,20]. As mentioned before, only one cohort study by Geier et al. examined this relationship [20]. Their findings showed that after adjusting for sex, age, and region of residence, patients with ASD had an aHR of 4.64, with a 95% CI of 3.63–5.93, compared with non-ASD controls. In their study, the effects of neuropsychiatric comorbidities were not considered. However, these comorbidities may have potential confounding effects. For example, the common neuropsychiatric comorbidity ADHD, which accounts for 35% of patients with ASD [12], has been reported to be positively associated with precocious puberty [21]. Therefore, the potential confounding effects of neuropsychiatric comorbidities, particularly ADHD, should not be ignored. In addition, based on the complex etiology between ASD and neuropsychiatric comorbidities, we also investigated the potential moderating effects of these comorbidities. The above considerations in our study design make our results more inferential.

In terms of the effect moderation by sex, the positive association between ASD and precocious puberty was more prominent in females on the additive scale, but not on the multiplicative scale. The inconsistent results between the additive and multiplicative scales can be explained by the fact that the incidence of precocious puberty in non-ASD patients differed significantly between females and males (1.73% versus 0.20%). In our study, females were nine times as likely as males to experience precocious puberty, which greatly increased the denominator of the ratio (HR_11_/(HR_10_ × HR_01_)) of multiplicative effect modification. Thus, even though the additive effect modification was strongly significant (RERI: 7.35), the multiplicative effect modification remained difficult to detect. Similar findings were reported and explained in the study by Vandenbroucke et al. [36]. Based on the previous literature, referring to multiplicative effect modification without considering the additive effect modification can lead to dangerous conclusions [36,37]. From a public health perspective, additive effect modification may be more relevant because it reflects the biological independence and stronger statistical power [37]. Therefore, our study implies that female patients with ASD may have a particularly high risk of precocious puberty.

We propose some possible mechanisms to explain the association between ASD and precocious puberty and effect modification by sex. In animal studies, Lee et al. demonstrated that D1 receptor overactivation or D2 receptor knockout induced autistic-like behaviors in mice [38]. The regulation of GnRH release, which is essential for puberty timing [39], has been linked to the dopamine system [40,41]. By acting on D1 receptors, dopamine stimulates the release of GnRH, and by acting on D2 receptors, dopamine inhibits the release of GnRH [40,41]. The sustained release of GnRH initiates puberty [42]. In addition, an animal study reported that the binding capacity of D2 receptors in the hypothalamus decreased during prepuberty in both sexes but was more pronounced in females [43]. This difference in D2 receptor activity may explain why females with ASD are more vulnerable to precocious puberty. However, these are only preliminary findings, and more human studies are warranted.

As for the effect moderation by neuropsychiatric disorders, we did not find effect heterogeneity across the strata defined by any specific neuropsychiatric disorder. This indicates that these comorbidities appear to have no direct impact on the association between ASD and precocious puberty. However, in the case of ASD comorbid with ADHD, there was a multiplicative rather than an additive effect modification. The negative-multiplicative null-additive effect modification by ADHD would be expected if the risk ratios for ASD alone (aHR 2.58) and ADHD alone (aHR 2.05) were comparable and if the ratio for ASD comorbid with ADHD (aHR 3.08) was only slightly higher than the ratios for ASD alone or ADHD alone [44]. In this situation, the joint effect of ADHD and ASD on precocious puberty was not salient enough to cause RERI positivity. Therefore, we took a more reserved attitude toward effect modification by ADHD.

In current clinical care, physicians typically focus on the emotional distress and interpersonal adjustment problems of adolescents with autism. However, there is less concern about the psychological impact of early puberty on these children. It is known that precocious puberty can cause a considerable mental burden in children, especially for girls [6]. For children with autism, early puberty may further exacerbate emotional and behavioral problems due to their poor adjustment to change. Therefore, the timely monitoring of pubertal development and the provision of relevant education and guidance to parents and children in advance may improve the quality of life for children with autism.

Our study has some limitations. First, due to the nature of the NHIRD, we were unable to obtain data on some important confounders, such as environmental hormone or toxin exposure, prenatal or perinatal events [45,46,47], and diet habits [48,49]. Second, we could not distinguish between central and peripheral precocious puberty in the ICD system. Finally, in the absence of laboratory and imaging examination, premature thelarche and premature adrenarche could not be distinguished from precocious puberty. The point estimates of the risk of precocious puberty may be overestimated.

## 5. Conclusions

In conclusion, our study suggests that patients with ASD are at a greater risk of precocious puberty, especially girls with ASD. Different comorbid neuropsychiatric disorders do not significantly change the association between ASD and precocious puberty. In clinical practice, sensitive and regular assessment of signs and symptoms of precocious puberty in patients with ASD will be important.

## Figures and Tables

**Table 1:** Demographic characteristics of ASD and non-ASD individuals.

Characteristics	ASD *n* = 29,320	Non-ASD *n* = 3,312,757
Age, mean (S.D) years	14.4 (3.6)	15.2 (4.0)
Sex, *n* (%)		
Male, *n* (%)	24,834 (84.70)	1,716,719 (51.82)
Female, *n* (%)	4486 (15.30)	1,596,038 (48.18)
Low income, *n* (%)	2858 (9.75)	320,928 (9.69)
**Neuropsychiatric comorbidity**		
Stringent ASD	24,102 (82.20)	-
ADHD, *n* (%)	18,801 (64.12)	167,880 (5.07)
Tics, *n* (%)	1797 (6.13)	23,668 (0.71)
OCD, *n* (%)	628 (2.14)	4269 (0.13)
Intellectual disability, *n* (%)	6301 (21.49)	26,848 (0.81)
Anxiety, *n* (%)	1779 (6.07)	25,843 (0.78)
Epilepsy, *n* (%)	1436 (4.90)	15,268 (0.46)
Precocious puberty, *n* (%)	353 (1.20)	30,988 (0.94)

ADHD, attention-deficit/hyperactivity disorder; ASD, autistic spectrum disorder; OCD, obsessive–compulsive disorder; *n*, number; S.D, standard deviation.

**Table 2:** Cox proportional hazard regression model analysis for risk of precocious puberty between ASD and non-ASD groups and effect modification by sex.

Group	Non-ASD Subgroup		ASD Subgroup		aHRs (95% CI) within Strata of Effect Modifiers	RERI (95% CI) Effect Modification on Additive Scale	aHR (95% CI) Effect Modification on Multiplicative Scale
	*n* (Precocious Puberty/Non-ASD with or without Effect Modifiers) (%)	aHR (95%CI)	*n* (Precocious Puberty/ASD with or without Effect Modifiers) (%)	aHR (95%CI)			
**ASD**	30,988/3,312,757 (0.94)	1.00	353/29,320 (1.20)	1.80 (1.61–2.01) *	-	-	-
Male	3351/1,716,719 (0.20)	1.00	136/24,834 (0.55)	1.73 (1.45, 2.06)	1.69 (1.40–2.03) *	-	-
Female	27,637/1,596,038 (1.73)	9.50 (9.16, 9.85)	217/4486 (4.84)	17.58 (15.24, 20.26)	1.86 (1.61–2.14) *	**7.35** (4.90, 9.80) *	1.07 (0.86, 1.33)
**Stringent ASD**	30,988/3,312,757 (0.94)	1.00	298/24,102 (1.24)	1.86 (1.64–2.09) *			
Male	3351/1,716,719 (0.20)	1.00	115/20,529 (0.56)	1.76 (1.46, 2.13)	1.72 (1.41–2.10) *		
Female	27,637/1,596,038 (1.73)	9.50 (9.16, 9.85)	183/3573 (5.12)	18.24 (15.64, 21.27)	1.93 (1.66–2.25) *	7.99 (5.23, 10.75) *	1.09 (0.86, 1.38)

Bold values and * denote statistical significance. aHR, adjusted hazard ratio; ASD, autistic spectrum disorder; CI, confidence interval; *n*, number; RERI, relative excess risk due to interaction.

**Table 3:** Cox proportional hazard regression model analysis for risk of precocious puberty between ASD and non-ASD groups and effect modification by different neuropsychiatric comorbidities.

Group		Non-ASD Subgroup		ASD Subgroup		aHRs (95% CI) within Strata of Effect Modifiers	RERI (95% CI) Effect Modification on Additive Scale	aHR (95% CI) Effect Modification on Multiplicative Scale
		*n* (Precocious Puberty/Non-ASD with or without Effect Modifiers) (%)	aHR (95%CI)	*n* (Precocious Puberty/ASD with or without Effect Modifiers) (%)	aHR (95%CI)			
ADHD	No	29,168/3,144,877 (0.93)	1.00	131/10,519 (1.25)	2.58 (2.17, 3.07)	2.41 (2.02–2.88) *		
	Yes	1820/167,880 (1.08)	2.05 (1.96, 2.16)	222/18,801 (1.18)	3.08 (2.69, 3.53)	1.57 (1.36–1.81) *	−0.56 (−1.17, 0.05)	**0.58** (0.46, 0.72) *
Tics	No	30,737/3,289,089 (0.93)	1.00	332/27,523 (1.21)	1.85 (1.65, 2.08)	1.81 (1.61–2.03) *		
	Yes	251/23,668 (1.06)	1.66 (1.46, 1.88)	21/1797 (1.17)	2.05 (1.33, 3.15)	2.13 (1.31–3.46) *	−0.47 (−1.39, 0.46)	0.67 (0.42, 1.05)
Intellectual disability	No	30,723/3,285,909 (0.93)	1.00	261/23,019 (1.13)	1.78 (1.56, 2.02)	1.74 (1.54–1.98) *		
	Yes	265/26,848 (0.99)	0.96 (0.85, 1.09)	92/6301 (1.46)	1.81 (1.47, 2.23)	2.13 (1.67–2.73) *	0.08 (−0.37, 0.52)	1.06 (0.81, 1.39)
Anxiety	No	30,576/3,286,914 (0.93)	1.00	316/27,541 (1.15)	1.81 (1.61, 2.03)	1.78 (1.59–2.01) *		
	Yes	412/25,843 (1.59)	1.24 (1.12, 1.38)	37/1779 (2.08)	2.18 (1.56, 3.04)	1.97 (1.37–2.84) *	0.13 (−0.62, 0.87)	0.97 (0.68, 1.38)
OCD	No	30,907/3,308,488 (0.93)	1.00	335/28,692 (1.17)	1.79 (1.60, 2.01)	1.78 (1.59–2.00) *		
	Yes	81/4269 (1.90)	1.41 (1.12, 1.78)	18/628 (2.87)	2.74 (1.72, 4.38)	2.81 (1.59–4.97) *	0.54 (−0.78, 1.86)	1.08 (0.64, 1.83)
Epilepsy	No	30,805/3,297,489 (0.93)	1.00	319/27,884 (1.14)	1.77 (1.58, 1.99)	1.77 (1.58–1.99) *		
	Yes	183/15,268 (1.20)	1.23 (1.06, 1.42)	34/1436 (2.37)	2.68 (1.90, 3.77)	2.17 (1.44–3.26) *	0.68 (−0.27, 1.62)	1.23 (0.84, 1.81)

Bold values and * denote statistical significance. ADHD, attention-deficit/hyperactivity disorder; aHR, adjusted hazard ratio; ASD, autistic spectrum disorder; CI, confidence interval; OCD, obsessive–compulsive disorder; *n*, number; RERI, relative excess risk due to interaction.

## Data Availability

Our study adopted data from the National Health Insurance Database. The database governs and stores all medical claims as an anonymous and encrypted dataset. These data are de-identified and cannot be used without application. To protect privacy, this database can only be accessed from a single site, the Data Science Centre, and the raw data are prohibited from being transferred to any portable storage device.

## Supplementary Materials

The following supporting information can be downloaded at: https://www.mdpi.com/article/10.3390/jpm14060632/s1, Table S1.
