# Supplementary material for: The Association between Autism Spectrum Disorder and Precocious Puberty: Considering Effect Modification by Sex and Neuropsychiatric Comorbidities
Source: J Pers Med. 2024 Jun 13;14(6):632. doi: 10.3390/jpm14060632 (PMC11204849; doi:10.3390/jpm14060632)
Supplement: Supplementary file 1 [file jpm-14-00632-s001.zip › jpm-3017104-supplementary.pdf]

Supplementary Table 1

Diseases	ICD-9CM	ICD-10
Primary hypothyroidism	244.9	E03.9
Central nervous system infection	320, 321, 322, 323, 324, 325, 326, 327	G00, G01, G02, G03, G04, G05, G06, G07
Congenital abnormalities of the central nervous system	741, 742	Q05, Q07
Septo-optic dysplasia	744	Q04
Tuberous sclerosis	759.5	Q85.1
Sturge-Weber syndrome	759.6	Q85.8, Q85.9
Ever radiation to the central nervous system	V58.0	Z51.0
